# Duplication and Evolution of *devA*-Like Genes in *Streptomyces* Has Resulted in Distinct Developmental Roles

**DOI:** 10.1371/journal.pone.0025049

**Published:** 2011-10-05

**Authors:** Laura C. Clark, Paul A. Hoskisson

**Affiliations:** Strathclyde Institute of Pharmacy and Biomedical Science, University of Strathclyde, Glasgow, United Kingdom; Charité-University Medicine Berlin, Germany

## Abstract

Understanding morphological transformations is essential to elucidating the evolution and developmental biology of many organisms. The Gram-positive soil bacterium, *Streptomyces coelicolor* has a complex lifecycle which lends itself well to such studies. We recently identified a transcriptional regulator, *devA*, which is required for correct sporulation in this organism, with mutants forming short, mis-septate aerial hyphae. *devA* is highly conserved within the *Streptomyces* genus along with a duplicate copy, *devE*. Disruption of *devE* indicates this gene also plays a role in sporulation; however the phenotype of a *devE* mutant differs from a *devA* mutant, forming long un-septate aerial hyphae. Transcriptional analysis of *devA* and *devE* indicates that they are expressed at different stages of the lifecycle. This suggests that following duplication they have diverged in regulation and function. Analysis of fully sequenced actinomycete genomes shows that *devA* is found in a single copy in morphologically simpler actinobacteria, suggesting that duplication has lead to increased morphological complexity. Complementation studies with *devA* from *Salinispora*, which sporulates but does not form aerial hyphae, indicates the ancestral gene cannot complement *devA* or *devE*, suggesting neo-functionalisation has occurred. Analysis of the synonymous and non-synonymous nucleotide changes within the *devA* paralogues suggest subfunctionalisation has occurred as both copies have diverged from the ancestral sequences. Divergence is also asymmetric with a higher level of functional constraint observed in the DNA binding domain compared with the effector binding/oligomerisation domain, suggesting diversification in the substrate specificity of these paralogues has contributed to their evolution.

## Introduction

The origin of biological novelty is a major theme in evolutionary and developmental biology and understanding key morphological transformations is paramount to elucidating these mechanisms. The differentiating soil bacterium *Streptomyces coelicolor* offers a genetically tractable model to study such morphological transitions due to its complex lifecycle, where a germinating spore gives rise to a colony of vegetative substrate mycelium. Vegetative growth proceeds by hyphal tip extension and by branching until changes in nutritional status [1] [2] and the accumulation of extracellular signalling molecules and surfactants [3], [4], [5], [6] trigger formation of specialised reproductive structures called aerial hyphae. These multigenomic aerial hyphae grow from the colony surface into the air, subsequently compartmentalising and maturing into unigenomic spores [2], [7], [8]. Mutants involved in this process can be classified into two broad groups: those blocked in their ability to form aerial hyphae (the *bld* mutants), and those able to form aerial hyphae but unable to complete their development into mature spores (the *whi* mutants).

The actinobacteria are a particularly diverse phylum both morphologically and physiologically and allow evolution of morphological complexity to be studied using phylogenomics and experimental studies. Isolation of mutants in the developmental process in the particularly well studied in *Streptomyces*, coupled with extensive genome sequencing and comparative genomics has shown that many of these genes fall into families with orthologues found throughout the phylum [8], [9].

The maintenance of large gene families in genomes is a heavily debated issue in both prokaryotes and eukaryotes [10]. In the actinomycetes, a general trend would appear that duplication of certain genes within the chromosome throughout evolution has contributed to developmental complexity; such as the Chaplins, Rodlins [11], [12], [13], *ssgA* [14], *whiJ/bldB* and *whiA/whiB* [8]. It is interesting to note that several of these genes are present in less-complex and non-sporulating actinomycetes with copy number broadly correlating with increasing developmental complexity suggesting that, through duplication and mutation, they have acquired sporulation specific roles [8]. The duplication of sequences in bacterial genomes is being identified more frequently and has now been demonstrated in a range of organisms [15], [16]. In *S. coelicolor* this also appears to be the case, with 709 genes having at least one homologue within the genome (with at least 70% sequence similarity, and 70% coverage on both proteins; Chandra, G., Personal communication). This is approximately 9% of the genome, which corresponds well with the published figures from other bacterial genomes [16], however extensive analysis of these genes is still required to confirm they are bona fide gene duplication events, rather than horizontal gene transfer (HGT) events, which are also known to contribute the expansion of gene families in bacteria [17].

Gene duplication is an important evolutionary force that provides an organism with an opportunity to evolve new functions. One or both of the duplicated genes can diverge to acquire differential regulation or mutations occur followed by evolution into a gene product with a new function. Duplication is also used as a mechanism to acquire a varied substrate spectrum. Thus, functional variations and differential regulation can be obtained as a result of gene duplication and provide an adaptive or fitness advantage in the natural environment. Indeed, data available for *Escherichia coli* and *Saccharomyces cerevisiae* suggest that gene duplication plays a key role in the growth of gene networks [18]. Classically, gene duplication is thought to enable duplicates to become specialised in different tissues or developmental stages [19]. Although a central issue developing from these observations is why so many duplicate genes have been retained in genomes even though the most likely fate of a redundant duplicate is non-functionalisation. The neofunctionalisation [19] and subfunctionalisation [20] models, however, are the most frequently used models to explain the retention of duplicates. The neofunctionalisation model postulates that the gain of new functions is the major selective factor for the retention of both duplicates in a genome. The subfunctionalisation model suggests that both duplicate genes undergo complementary degeneration, so that both copies are required to fully complement the ancestral gene and can be considered an essentially non-adaptive process. Studies of yeast paralogues suggest that both copies of duplicate genes become more specialised in their expression, and that neofunctionalisation is more common than subfunctionalisation [21]. However, it is also possible for both mechanisms to work in parallel, with a large proportion of genes undergoing rapid subfunctionalisation following duplication, followed by a prolonged period of neofunctionalisation [22]. Gene duplication is therefore an important prerequisite for gene innovation, facilitating adaptation with paralogues comprising an increasingly recognised proportion of bacterial genomes. This importance to biological innovation is likely to contribute to the evolution of complex lifecycles in actinobacteria, given the observed numbers of paralogous gene families associated with development and sporulation in complex actinobacteria, as it has previously been observed that increasing gene family size often correlates with increasing developmental complexity [23].

Here we demonstrate that the duplication of a recently identified metabolite responsive transcriptional regulator in *Streptomyces coelicolor* has lead to evolution of novel functions in each paralogue. DevA is a member of the GntR family of proteins, which controls the expression of itself and a putative phosphatase (*devB*) through negative autoregulation [24]. Adjacent to *devA* on the chromosome is a homologue of *devA*, *devE*, which has arisen through gene duplication. These regulators, both essential for correct development and have diverged from an ancestral homologue in developmentally less complex actinomycetes, demonstrating neo and sub-functionalisation during the transition such that the ancestral gene cannot complement their function.

## Results

### *devA* and *devE* are paralogous regulators which have distinct roles in the development of streptomycetes

We recently identified a gene encoding a GntR-like regulator, *devA*, in *S. coelicolor* which upon disruption had profound effects on the formation of aerial hyphae and [24]. Located adjacent to *devA* (SCO4190) on the *S. coelicolor* chromosome is a duplicate, designated *devE* (SCO4188; [24]). *devE* encodes a 303 amino acid protein which is predicted to be a GntR-like regulator, with a putative helix-turn-helix motif at residues 46–67 (Score 6.60; 2, 12, 25). DevE shows 57.6% identity with DevA and both genes are conserved in *S. avermitilis* and *S scabies* [24].

**Figure 1:**
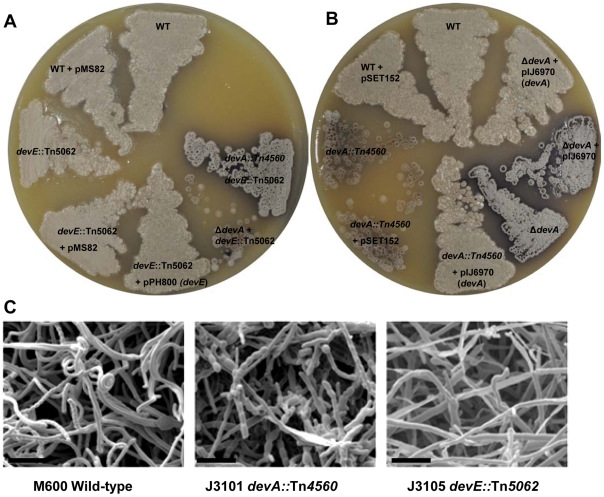
Deletion of *devA* or *devE* results in aberrant sporulation in *Streptomyces coelicolor*. **A:** Effect of *devA* & *devE* disruptions on colony appearance and complementation of mutants. Strains were grown on MS medium for 5 days. **B:** Scanning electron microscopy images of *devA* and *devE* mutants grown on MS medium. SEM Bar = 10 µm.

It was hypothesised that, if *devA* is required for correct sporulation then a mutation in the paralogous *devE* gene would also result in defective development. Therefore, we created a Tn*5062* insertion in *devE* (J3113) to investigate its phenotype (**Fig. 1A**). The *devE* mutant is indeed defective in development, however morphologically different to a *devA* mutant; forming aerial hyphae at normal levels which fail to septate and give rise to spores (**Fig. 1C**). This is in contrast to the short, mis-septate aerial hyphae of a *devA* mutant, which results in a *whi* colony phenotype (**Fig. 1B and 1C**). These data suggest that DevA and DevE have distinct roles in the *Streptomyces* developmental process. To ensure there was no cross regulation between *devA* and *devE*, a *devA* null mutant was created using an oligonucleotide co-electroporation approach (see materials and methods). This approach will likely result in the constitutive expression of *devB*, a putative phosphatase, due to the lack of a *devA* coding sequence, whose gene product has previously been shown to negatively autoregulate its own transcription [24]. The Δ*devA* mutant (J3106) exhibits a white (*whi*) phenotype (**Fig. 1B**) which is fully complemented by a wild-type copy of *devA* on an integrating plasmid, pIJ6970 (**Table 1**), suggesting there is no cross regulation of *devA* by *devE*. This is further supported by the observation in Hoskisson *et al.*, [24] of an increase in transcription from the *devA* promoter in a *devA* mutant, due to the lack of auto-regulation. Thus is *devE* bound the *devA* promoter this increase in transcription would not be observed.

**Table 1:** Strains and plasmids used in this study.

Strain or plasmid	Genotype/comments	Source or reference
**Strains**		
***S. coelicolor***		
M600	Prototrophic, SCP1^−^ SCP2^−^	[47]
J3101	M600 *devA*::Tn*4560* (viomycin)	[24]
J3102	M600 *devA*::Tn*5062* (Apramycin)	[24]
J3105	M600 *devE*::Tn*5062* (Apramycin)	This work
J3106	M600 Δ*devA*, in-frame deletion of *devA* CDS	This work
sPH101	M600 *devA*::Tn*4560/ devE*::Tn*5062* (Apramycin and viomycin)	This work
sPH102	M600 Δ*devA/devE::*Tn*5062* (Apramycin)	This work
**Plasmids**		
pIJ6902	Plasmid integrating at phage φC31 *attB* site, carrying apramycin resistance (*apr*) and thiostrepton resistance (*thio*) with thiostrepton inducible promoter (*tipA*)	[26]
pIJ6970	pSET152 carrying 1.5-kb *devA* fragment	[24]
pMS82	Plasmid integrating at phage φBT1 *attB* site, carrying hygromycin resistance (*hyg*)	[52]
pPH800	Plasmid integrating at phage φBT1 *attB* site, carrying hygromycin resistance (*hyg*) containing the 1.2-kb *devE* fragment.	This work
pPH801	Plasmid derivative of pIJ6902 integrating at phage φC31 *attB* site, carrying apramycin resistance (*apr*) and thiostrepton resistance (*thio*) with thiostrepton inducible promoter (*tipA*) driving expression of *devA*_sal_	This work
pPH802	Plasmid derivative of pIJ6902 integrating at phage φC31 *attB* site, carrying apramycin resistance (*apr*) and thiostrepton resistance (*thio*) with thiostrepton inducible promoter (*tipA*) driving expression of *devA* from *S. coelicolor*.	This work
pPH803	Plasmid derivative of pIJ6902 integrating at phage φC31 *attB* site, carrying apramycin resistance (*apr*) and thiostrepton resistance (*thio*) with thiostrepton inducible promoter (*tipA*) driving expression of *devE* from *S. coelicolor*.	This work

The creation of a *devA/devE* double mutant (**Fig. 1A**) does not appear to affect the phenotype beyond that of a single mutant, when a copy of cosmid D66 Tn*5062*::*devE* (apramycin resistant) is introduced in to either a Δ*devA* (J3106:unmarked) or a Tn*4560*::*devA* (J3101:viomycin resistan) background. This again suggests that these genes have diverged in function and do not cross-complement each other and have separate temporal roles in development of *S. coelicolor*.

### *devA* and *devE* are expressed at different stages of the lifecycle in *S. coelicolor*

**Figure 2:**
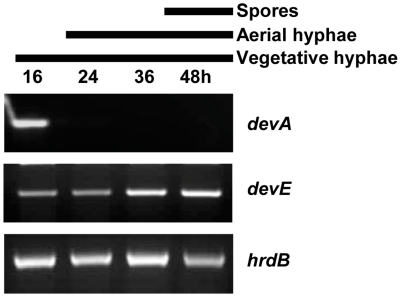
Transcriptional analysis of *devA* & *devE*. RT-PCR of *devA* and *devE* during development of *S. coelicolor* M600 on MS medium. The time points at which mycelium were harvested for RNA and the developmental stage of the culture, as judged by microscopic examination, are shown above.

Evolutionary modifications of gene expression are considered one of the platforms from which morphological diversification has arisen (Prud'homme *et al.*, 2007). It has previously been shown by S1 nuclease mapping that *S. coelicolor devA* [24] is actively transcribed until about 24 hours of growth on solid medium. Given that divergence in expression patterns is important for new gene functions to emerge from duplicates, the expression of *devE* throughout the lifecycle of *S coelicolor* was investigated (**Fig. 2**). Semi-quantitative RT-PCR shows that transcription of *devE* is continuous throughout growth however it does show an increase during spore formation relative to the multiplexed vegetative sigma factor *hrdB*. RT-PCR of *devA* using the same RNA time course showed the transcript is present up to 16 hrs of growth confirming previous data [24]. Microarray data from *S. coelicolor* grown on minimal medium and on rich medium also confirms this observation of differential transcription (C.M. Kao, Personal communication). The expression of *devE* later in growth under the same conditions, during the onset of septation is consistent with the morphological phenotypes observed. *devA* and *devE* are temporally separated during development and this reflects their activity in two aspects of development; erection of aerial hyphae (*devA*) and septation of aerial hyphae (*devE*). Thus, following duplication, altered regulation of these two genes is likely to have contributed to their divergence.

### The DevA subfamily is duplicated in aerial hyphae forming and sporulating actinomycetes

**Figure 3:**
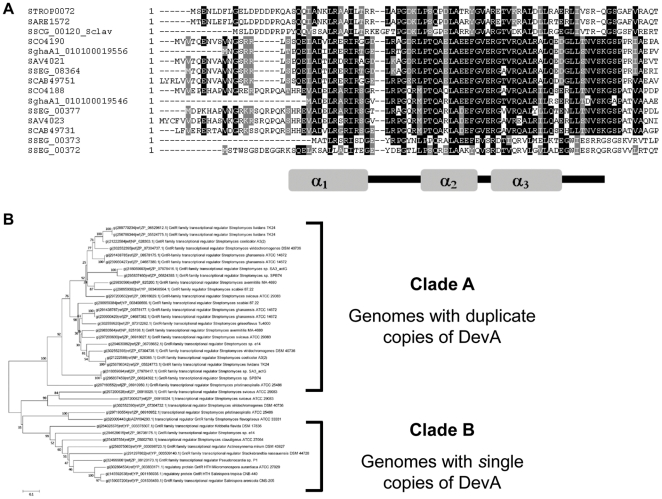
Bioinformatic and evolutionary analysis of *devA* and its homologues. **A:** Multiple alignment of the N-terminus of the DevA-like protein sequences. The α-helices (1–3) of the helix-turn-helix motif are shown to indicate the structure based homology. **B:** A ML tree based on the alignment of whole DevA-like sequences displaying the two main clades (A - duplicates & B - singletons). Please see Table 2 for details on homology and morphology of the strains.

A significant number of homologues were identified by using the DevA sequence to interrogate the non-redundant database (BLAST; E-value<1^−10^, BLAST Scores>100). This is higher than that reported in Hoskisson *et al.*, [24] likely as a result of increased actinobacterial genome sequencing. Analysis and reciprocal BLAST best-hits of homologues identified during the search, confirmed the presence of *devA*-like genes in morphologically diverse actinomycetes (**Fig. 3A/3B**). *Streptomyces* genomes largely contain at least two paralogues of *devA*, with a few exceptions, such as *S. clavuligerus* and *S. griseus* which may suggest some degree of niche specialisation through the addition developmental checkpoints.

Extensive analysis of the sequences indicate a highly conserved N-terminal helix-turn-helix domain (**Fig. 3A**) showing high degrees of similarity in the structurally conserved α-helix regions (α_1–3_). The C-terminal effector binding/oligomerisation domain (Eb/O) also shows high degrees of conservation characteristic of the DevA-subfamily [24], [25].

BLAST analysis reveals that in species containing two copies of DevA-like proteins there is a paralogue with high amino acid identity with DevA (SCO4190) and one with more distant homology, in close proximity on the chromosome suggesting there has been divergence following a duplication event (**Table 2**).

**Table 2:** Homology and Morphology of selected strains containing *devA*-like genes.

Organism /homologue	Developmental morphology	% identity to *DevA* (SCO4190)	% identity to *DevE* (SCO4188)	Reference
***Streptomyces coelicolor –*** **DevA (SCO4190)**	Filamentous, aerial hyphae & spores	-	58	[24], [53]
***Streptomyces coelicolor –*** **DevE (SCO4188)**	Filamentous, aerial hyphae & spores	57	-	[24], [53]
***Streptomyces ghanaensis –*** **SghaA1010100019556**	Filamentous, aerial hyphae & spores	77	78	[53]
***Streptomyces ghanaensis –*** ** SghaA1010100019546**	Filamentous, aerial hyphae & spores	59	77	[53]
***Streptomyces avermitilis –*** **SAV4021**	Filamentous, aerial hyphae & spores	76	60	[53]
***Streptomyces avermitilis –*** **SAV4023**	Filamentous, aerial hyphae & spores	56	75	[53]
***Streptomyces sviceus –*** **SSEG08364**	Filamentous, aerial hyphae & spores	74	59	[53]
***Streptomyces sviceus –*** ** SSEG00377**	Filamentous, aerial hyphae & spores	60	78	[53]
***Streptomyces sviceus –*** ** SSEG00373**	Filamentous, aerial hyphae & spores	33	29	[53]
***Streptomyces sviceus –*** ** SSEG00372**	Filamentous, aerial hyphae & spores	31	31	[53]
***Streptomyces scabies –*** **SCAB49751**	Filamentous, aerial hyphae & spores	74	58	[53]
***Streptomyces scabies –*** ** SCAB49731**	Filamentous, aerial hyphae & spores	59	71	[53]
***Streptomyces clavuligerus –*** **SSCG00120**	Filamentous, aerial hyphae & spores	33	33	[53]
***Salinispora tropica –*** **STROP0072**	Filamentous, single spores on vegetative hyphae	35	34	[54]
***Salinispora arenicola –*** **SARE1572**	Filamentous, single spores on vegetative hyphae	35	34	[54]
***Kribella flavida –*** **KflaDRAFT6164**	Filamentous fragmenting, aerial hyphae formed	33	34	[55]
***Stackebrandtia nassauensis –*** **SnasDRAFT27490**	Filamentous fragmenting , aerial hyphae formed	32	31	[56]
***Actinosynnema mirum –*** ** AmirDRAFT51580**	Filamentous fragmenting, aerial hyphae formed, motile spores	35	30	[57]

**Figure 4:**
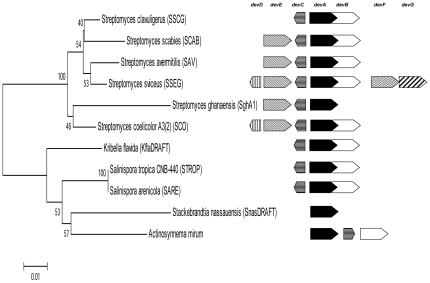
A maximum likelihood tree based on the alignment of the 16S rRNA gene of selected *devA*-like containing species coupled with the *devA* gene context of each species indicating the duplication event in the *Streptomyces* lineage.

Phylogenetic analysis of the sequences (**Fig. 3B**) confirms that the sequences are divided in to two distinct lineages, which is consistent whether the whole protein sequence (Data not shown), HTH domain (**Fig. 3A**) or Eb/O (Data not shown) are analysed. *devA*-like genes largely display the same genetic context (**Fig. 4**), with *devA* being co-transcribed with the putative phosphatase/hydrolase (DevB). Divergently transcribed is the *devC* gene that encodes a small hypothetical protein of approximately 50 amino acids. The duplicated *devA*-like gene, *devE*, is located upstream, but the duplication only appears to have maintained the GntR-like regulator, with the *devC*-like gene only being maintained in *S. sviceus* and *S. coelicolor*.

The DevA lineage split largely correlates with the developmental phenotypes observed within the group (**Fig. 3B** **;** **Table 2**). Phylogenetic analysis of the sequences using neighbour-joining (NJ; Saitou and Nei, 1987) and maximum Likelihood (ML; Tamura *et al.*, 2007) trees showed highly similar topologies enhancing confidence in the trees obtained. This analysis showed that Clade A is formed entirely from *Streptomyces* species which undergo a complex developmental cycle with the formation of aerial hyphae followed by septation and the formation of mature spores. This clade can be further divided in to two sub-groups, each one consisting of a single homologue from each organism, those closest to DevA and those closest to DevE. This clearly indicates that a duplication event has occurred from an ancestral DevA-like gene and has subsequently diverged resulting in the observed tree topology. Clade B consists of actinobacteria with less complex lifecycles and morphologies. Those which fragment their hyphae rather than forming true spores (*Kribella*), those which form aerial hyphae but not spores (*Stackebrandtia*, *Actinosynnema*) or species which form single spores directly on the vegetative mycelium (*Salinispora*, *Micromonospora*) (**Table 2**). One exception to this is *Streptomyces clavuligerus*, which only has one copy of the *devA*-like genes, suggesting that maintenance of the duplication has not occurred in this species. There is no characterisation of these genes in other streptomycetes, however the maintenance of such gene duplication events is likely to be highly niche specific, and understanding the exact micro-niches occupied by such a highly speciated genus as this suggests different selective pressures acting on developmental checkpoints.

*S. sviceus* has four copies of *devA*-like genes on the chromosome, suggesting multiple duplication events may have occurred in this species or they have acquired additional copies through HGT which may reflect the association with Clade B in some strains such as *S. sviceus* (SSEG00372; DevF and SSEG00373; DevG; **Fig. 3B**).

### *devA* from *Salinispora* cannot complement either a *devA* or a *devE* mutant in *Streptomyces*

**Figure 5:**
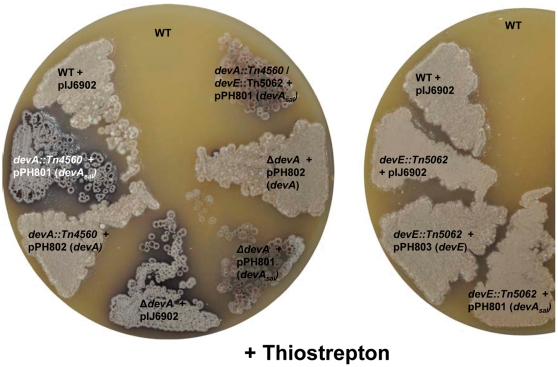
Effect of complementation of *devA* and *devE* disruptions with *devA* from *Salinispora tropica*, under the control of the thiostrepton inducible promoter (*tipA*), on colony appearance. Strains were grown on MS medium for 5 days in the presence of thiostrepton.

The different roles played in development by these genes in *S. coelicolor* suggest that their duplication has resulted in divergence and sub- or neo-functionalisation. To test if this divergence is neo-functionalisation (change in function) or sub-functionalisation (division of ancestral function between the duplicates), the *devA* gene of *Salinispora tropica* was cloned into pIJ6902 [26] under the control of the *tipA* promoter (pPH801). The *tipA* promoter was used to ensure that differences in promoter recognition of RNA-polymerase between *Streptomyces* and *Salinispora* did not affect transcription of the gene. In addition both *devA* (pPH802) and *devE* (pPH803) from *S. coelicolor* were cloned into pIJ6902, and examined to ensure that driving these genes from the *tipA* promoter resulted in complementation of the mutant phenotype. All strains were tested in the presence (**Fig. 5**) and absence of the inducer, thiostrepton (data not shown). Introduction of the *devA_sal_* construct into either a *devA* or a *devE* mutant did not complement the function of these genes in *S. coelicolor* (**Fig. 5**). The introduction of pPH801 in to the *devA/devE* double mutant also did not complement the lesions in these strains (**Fig. 5**). Driving the *S. coelicolor devA* (pPH802) and *devE* (pPH803) from the *tipA* promoter (**Fig. 5**) in each mutant background resulted in complementation. This indicates that divergence following the duplication event has been sufficient to render the ancestral homologue incapable of complementation in the duplicate copies in *Streptomyces*, suggesting that neo-functionalisation has occurred in both duplicates in *Streptomyces*.

### Evolutionary and functional constraints on *devA*-like genes

The apparent neo-functionalisation of the *devA*-like genes in *Streptomyces* raises interesting questions regarding the process of divergence of these genes in actinomycetes; does the neo-functionalisation model adequately explain the inability to complement both genetic lesions? To test this we aligned the nucleotide sequences of the *devA*-like genes and calculated the number of non-synonymous substitutions per non-synonymous site within the sequences (dN). These are all <1 (mean = 0.4), suggesting a functional constraint upon these proteins. Interestingly when these values are calculated for each of the clades, the dN value differs between each group, with a lower number of non-synonymous substitutions per non-synonymous site being observed in Clade A, containing the species with duplicated genes.

To further investigate the levels of selection imposed on these genes the ratio of non-synonymous substitution to synonymous substitutions per site was calculated [27]. All proteins exhibit a dN/dS ratio of <1 indicating that purifying selection is constraining these genes, through the removal of non-synonymous mutations (**Table 3**). There is however a marked difference when Clade A and Clade B are compared, as for dN (**Table 3**) indicating that selection is acting differently upon the species containing one or two copies of a *devA* homologue, although standard deviations of these data suggest it may not be significant. The calculation of dN and dN/dS for *devA* and *devE* homologues separately however indicates that purifying selection is acting to remove non-synonymous mutations and constraining function in duplicates and is significant when compared to Clade B (singleton) *devA* homologues.

**Table 3:** Evolutionary analysis of DevA-like homologues.

*Sequence group*	*dN*	dN/dS
All *devA* homologues	0.40 (+/−0.05)	0.13 (+/−0.24)
Clade A - (*devA/E* duplicates)	0.25 (+/−0.04)	0.57 (+/−0.42)
Clade B - (*devA* singletons)	0.55 (+/−0.06)	0.86 (+/−0.33)
Clade A - (*devA* paralogues only)	0.25 (+/−0.04)	0.20 (+/−0.13)
Clade A - (*devE* paralogues only)	0.25 (+/−0.04)	0.12 (+/−0.12)
All *devA* homologues - N-terminal domain	0.35 (+/−0.18)	0.68 (+/−0.40)
All *devA* homologues -C-terminal domain	0.39 (+/−0.15)	0.95 (+/−0.20)

To gain an understanding of how rapid the divergence between DevA homologues has been, the synonymous substitutions per site were calculated between *devA* (0.15+/−0.03) and the *devE* paralogues (0.33+/−0.04), suggesting a higher rate of divergence for *devE* following the duplication event, consistent with this being the duplicated gene.

Division of the proteins into their domains (N-terminal HTH and C-terminal Eb/O) and then recalculation of the dN/dS ratio for each of the domains revealed that the purifying selection is higher on the N-terminal HTH domain in both clades, suggesting that maintenance of DNA binding ability is a key selective pressure on these genes (**Table 3**). Relaxed selective pressure on the C-terminal domain is indicative of functional divergence by diversifying the Eb/O domain at a faster rate than DNA binding, perhaps broadening the metabolite binding capabilities.

## Discussion

Bacteria can adapt to selective conditions by either altering regulatory responses to the environment, by acquisition of stable, adaptive mutations or through gene duplication-amplification, which alters gene dosage and copy number [15]. Gene duplication is a major source of biological novelty throughout the three kingdoms of life and can facilitate adaptation through the acquisition of novel functions. Here we have shown that duplication of a regulatory protein in actinomycete bacteria has contributed to the increased complexity of the developmental process in these organisms through duplication and sequence divergence.

The HTH containing GntR family is widely distributed throughout the bacteria, where they regulate diverse biological processes. In general, these proteins contain a DNA-binding HTH domain at the N-terminus and an Eb/O domain at the C-terminus [25]. Upon binding of an effector molecule at the C-terminal domain, a conformational change occurs in the protein dimer which influences the DNA-binding properties of the HTH domain of the protein, altering transcription at its cognate promoters. The DNA-binding domain is conserved throughout the GntR family [28], with the regions outside the DNA-binding domain being more variable [25]. Often these proteins are negatively autoregulatory and this ability to tightly control gene expression in response to metabolites allows cells to respond to the environmental conditions and physiological state of the cell. In developing organisms the commitment to differentiate is tightly regulated to ensure that the profound cellular consequences of the process are not undertaken during transient environmental changes. In *Streptomyces* six GntR regulators are known to be involved in the regulation of sporulation [24], [29], [30], [31], [32], indicating that sensing and responding to metabolic changes is fundamental to regulating this process.

### Phenotypic and regulatory divergence between *devA* and *devE*

The presence of two copies of *devA*-like genes in *Streptomyces*, which have different functions in development and exhibit different expression patterns indicates that the novel function evolved following duplication in these genes, given the inability of a pre-duplication copy of *devA* (*Salinispora*) to complement the genetic lesions in *Streptomyces*. The *devA* mutant [24] forms spores and short aberrant aerial hyphae, suggesting that this gene may be responsible for sensing a metabolite during growth of aerial hyphae and may signal when aerial hyphae extension should stop. The *devE* mutant forms normal length aerial hyphae which fail to curl or septate, suggesting that metabolite sensing plays a role in the initiation of septum formation. Our understanding of the functions, regulons and metabolites sensed by these proteins is still in its early stages, however we know of at least one gene controlled by DevA, a putative phosphatase/hydrolase, *devB* [24] which also exhibits a developmental phenotype. A *devB* mutant is conditionally bald and here we have shown through deletion of the complete *devA* coding sequence, that constitutive expression throughout out the lifecycle results in the inability to form aerial hyphae. This suggests that tight regulation of this putative phosphatase/hydrolase is required for correct sporulation. How this fits in to the wider developmental hierarchy is currently unknown.

Following the duplication event these genes have also diverged in terms of their transcription, *devA* is expressed early in growth, correlating with the observed phenotype of shortened aerial hyphae and *devE* is expressed throughout growth, although apparently in increased amounts when aerial hyphae and spores are present. The divergence of expression has long been considered a key step in the emergence of a new gene from a duplicate copy [33] and may lead to tissue specific expression as we have observed here.

### Paralogous regulators found in aerial hyphae forming and sporulating actinomycetes

Sporulation is an adaptive process allowing survival under sub-optimal growth conditions [34]. The *devA*-like genes have only been found in actinomycetes that differentiate [24]. The duplication event and its subsequent preservation in *Streptomyces* indicate that both copies of the *devA*-like genes are performing a specific function in this group, observed by the different phenotypes in the mutants and their differential transcription. The origin of this gene is difficult to ascertain through extensive PSI-BLAST searching and sequence analysis (Data not shown). However, the distribution throughout several actinobacterial sub-orders (*Glycomycineae*, *Micromonosporineae*, *Proprionibacterineae*, *Pseudonocardineae*, *Streptomycineae*) suggests it may have been lost in some lineages within the *actinomycetales* order (based on the 16S phylogeny in Stackebrandt et al., [35], with several well studied sub-orders not containing hyphae forming or sporulating species or copies of *devA*-like genes. However extensive genome sequencing has not been undertaken in many sub-orders, mainly due to a lack of medical or industrial interest and further genomes and ecological studies may allow increased insight in to the evolution of the developmental process in this group and the possible roles that certain metabolite responsive proteins such as GntR regulators may play in niche specialisation.

### The *devA* subfamily in *Streptomyces* has undergone subfunctionalisation followed by neofunctionalisation

Evolutionary analysis of the *devA* subfamily indicates that subfunctionalisation occurred initially following the duplication event, probably at the emergence of the *Streptomycinae*, given the different gene expression profiles observed and the conservation of both duplicates within the lineage. The levels of purifying selection identified by the dN/dS ratio (<1) indicates a functional constraint maintaining these genes, however it is known that proteins with dN/dS<1 may still contain sites under positive selection [36]. The different values obtained for synonymous changes per synonymous site (dS) for the paralogous *devA* and *devE* groups indicate that subfunctionalisation is occurring as both genes are diverging from each other, fitting with the subfunctionalisation model, which predicts that two genes with identical functions and regulation are unlikely to be maintained in a genome [22], [37], [38]. Additionally the *devA* and *devE* mutants exhibit different phenotypes and neither is complemented by the ancestral copy of *devA* from *Salinispora* which in consistent with the neofunctionalisation model of duplicate fates [20], [39].

Comparison of dN/dS ratio of each domain suggests there is an asymmetric functional constraint on each domain of *DevA*, with purifying selection acting stronger on the N-terminal HTH domain than on the C-terminal Eb/O domain. The constraint therefore acts upon DNA binding domain more strongly, maintaining the regulatory role of the protein while freeing selection of the Eb/O domain, which can potentially evolve novel effector binding capabilities allowing responses to more diverse substrates.

Subfunctionalisation followed by neofunctionalisation is not unprecedented and appears to explain the evolution of many duplicate genes due to relaxed constraint following duplication in plants and fungi [40], [41], [42]. It remains to be seen if duplication is a major route to gene innovation in prokaryotes, given the importance of horizontal gene transfer in these organisms [17], yet the evolution of large gene families in higher organisms has established these models of evolution and bacterial systems exhibiting these processes offer unique, tractable model systems to understand these processes in molecular detail.

## Materials and Methods

### Bacterial strains, plasmids, growth conditions and conjugal transfer from *E. coli* to *Streptomyces*

The *S. coelicolor* strains used in this study are summarised in **Table 1**. All strains were cultivated on minimal medium (MM) containing mannitol (0.5% w/v) or mannitol and soya flour (MS) agar [43]. Conjugation of plasmids from the *E. coli* strain ET12567 (*dam dcm hsdS*), containing the driver plasmid pUZ8002, was used to bypass the methyl-specific restriction system of *S. coelicolor* [44].

### Construction of a *devA* null mutant, a *devE* and a *devAE* double mutant

A derivative of cosmid D66 carrying Tn*5062* insertion in *devE* (gifts of Dr Lorena Fernández-Martínez and Professor Paul Dyson, University of Swansea) generated using the *in vitro* transposition method of Bishop *et al.* [45], was introduced into *S. coelicolor* M600 by conjugation from *E. coli* ET12567/pUZ8002. Mutants exhibiting the double-crossover phenotype (apramycin resistant, kanamycin sensitive) were confirmed by Southern hybridisation and designated J3105 (*devE*::Tn*5062*).

Construction of a *devA* in-frame deletion null mutant was achieved by PCR-targeting of linearised cosmid D66 in λ-RED-proficient *E. coli*, using the method of Gust *et al.* [46] as partially described in [24]. Briefly, the rare cutting *Afl*II site in Tn*5062* (27 sites in the *S. coelicolor* genome) was utilised to remove the complete *devA* coding sequence. A derivative of cosmid D66 carrying a Tn*5062* insertion in *devA* was linearised within the transposon by digestion with *Afl*II (the parent D66 cosmid contains no *Afl*II sites). Uncut cosmid was eliminated by gel electrophoresis and the linearised cosmid was co-electroporated into BW25113/pIJ790 along with a 100-mer oligonucleotide (5**′**-AAACAAGTTTCAAACAACTCCCTATAGGTAGGTCGAAGTTGTAGCGTTTGATCACAGAAGTGGTTCGACGCCCTCTGGGAAACCATCACCACGGACATGA-3′), consisting of two 50-nt sequences corresponding to the upstream and downstream regions of the *devA* gene (leaving the desired deletion junction underlined above). Re-circularisation of the cosmid was brought about by double crossing over between the 5′- and the 3′- ends of the oligonucleotide and the linearised cosmid, resulting in colonies resistant to kanamycin (cosmid marker) and sensitive to apramycin (carried by Tn*5062*, confirming deletion of the transposon). The mutant cosmid D66 (D66DeltadevA) was confirmed by sequencing [24]. To introduce the null allele into *S. coelicolor* the *devA* mutant (J3102) was protoplasted according to Kieser *et al.* [47] and these were transformed with the mutant cosmid lacking the *devA* coding sequence (D66Delta*devA*). Single-crossover mutants were selected on kanamycin and subsequent double crossover (*devA* null) mutants were selected following a round of growth on non-selective media and replication to apramycin and kanamycin to confirm loss of the cosmid. The chromosomal location of the *devA* null mutant was confirmed by sequencing and verified by Southern blotting. This strain was designated J3106.

The original *devA* mutant, J3101 was used to create a double *devAE* mutant as follows: the *devE::*Tn*5062* derivative of cosmid D66 used above was introduced in to the viomycin resistant J3101 (*devA:*Tn*4560*; [24]) and double cross-over mutants were selected using apramycin and viomycin resistance and kanamycin sensitivity, to ensure both transposons were maintained in the mutants, avoiding homogenitization of *devA* with a wild-type copy of *devA*. This strain was designated sPH101. An additional *devAE* double mutant was created by introducing the *devE::*Tn*5062* derivative of cosmid D66 in to J3106 to create a *devAE* double mutant which ensured that no polar effects were observed on *devB*. The absence of *devA* in the double-crossovers was checked by PCR using the primers used in the RT-PCR reactions for *devA* (Data not shown). This strain was designated sPH102.

### Plasmid construction

Plasmids used in this work are described in **Table 1**. Plasmids were constructed as follows. pPH800: a 1.2-kb fragment carrying *devE* was amplified from cosmid D66 using oligonucleotides 5′-GCCCGTACTTCCACTGCA -3′ and 5′-CCAAGAGCCCCTCCGTCA-3′ and ligated into the *Eco*RV site of pMS82. pPH801: an 880-bp fragment carrying the *devA_Sal_* was amplified from *Salinispora tropica* (DSM 44818) genomic DNA using the oligonucleotides 5′- GGGCATATGAGCGAGAACCTTGACTT-3′ (containing an engineered NdeI site) and 5′- CTGAATTCTCATGTGTCGTACCGGT-3′ (containing an engineered EcoRI site) and was cloned in to pGEM-T-Easy (Promega) according to the manufacturers instructions. The fragment was sequenced to confirm its identity. The 880 bp fragment was excised using NdeI and EcoRI and subcloned in to pIJ6902 cut with NdeI and EcoRI, resulting in *devA_Sal_* being cloned upstream of the Thiostrepton- inducible promoter *tipA*, this plasmid was named pPH801.

To ensure that *devA* and *devE* can complement their corresponding mutants when expressed from a *tipA* promoter, both sequences were cloned in to pIJ6902 and introduced in to the appropriate strains. The *devA* sequence was subcloned from a pGEM-T-Easy derivative, containing *devA* with an engineered 5′-NdeI site [24], in to pIJ6902, resulting in pPH802. The *devE* sequence was amplified by PCR from cosmid D66 using oligonucleotides 5′-CATATGGTCGTGGTTCGACGC-3′ (containing an engineered NdeI site) and 5′-TGGGCGAGGGCGGACTGAGCTC-3′ and cloned in to pGEM-T-Easy. The fragment was subcloned, using NdeI (oligonucleotide) and EcoRI (in pGEM-T-Easy), in to pIJ6902 resulting in pPH803.

### RNA isolation RT-PCR of *devA* and *devE*

RNA samples were isolated throughout the lifecycle of wild-type and mutant strains of *S. coelicolor* as previously described [24].The Qiagen One-Step RT-PCR kit was used to amplify sequences of interest according to the manufacturers instructions, using 25 cycles of amplification. The following primers were used for amplification of *devA* (forward 5′-GAGGAGTTCGGCGTGGA-3′; Reverse 5′- AGCCGAGCGCGTCGTA-3′), for *devE* (forward 5′-TCGACGCGCTCTGCCTGA-3′; Reverse 5′-TCCCCCACAGTGCGTCGA-3′) and the vegetative sigma factor *hrdB* was used as a control in a multiplex PCR for constitutive expression and amplification using the following primers (forward 5′-GAGGCGACCGAGGAGCCGAA-3′; Reverse 5′-GCGGAGGTTGGCCTCCAGCA-3′).

### Microscopy

Light microscopy and scanning electron microscopy were performed as described previously [48].

### Sequence alignment and Phylogenomic analysis

All predicted protein sequences and nucleotide sequences were downloaded from the NCBI database (www.ncbi.nlm.nih.gov). Homologous sequences were identified by BLASTP against the non-redundant protein sequence database using DevA from *Streptomyces coelicolor* as a query. Paralogues and orthologues were confirmed by reciprocal best hit BLAST searching between the genomes.

Alignments of DevA orthologues were generated using ClustalW [49] with default options. Phylogenetic trees were reconstructed using neighbour-joining (NJ; [50]) and maximum Likelihood (ML) with default parameters as implemented in MEGA 4.0 [27]. The reliability of these trees was estimated by the bootstrapping with 1000 replicates.

The number of synonymous nucleotide substitutions (dS) to non-synonymous nucleotide substitutions (dN) and the ratio of synonymous nucleotide substitutions and nonsynonymous nucleotide substitutions (dN/dS) were calculated by the model of modified Nei-Gojobori method [51], applying the Jukes-Cantor corrections in the MEGA 4.0 software suite [27].

The 16S rRNA genes were downloaded from the NCBI database (www.ncbi.nlm.nih.gov) and they were aligned using ClustalW with phylogenetic reconstruction performed using NJ and ML methods in MEGA 4.0 [27], [49].
